# Role of LINC01592 in TGF-β1-induced epithelial-mesenchymal transition of retinal pigment epithelial cells

**DOI:** 10.18632/aging.203023

**Published:** 2021-05-25

**Authors:** Ying Su, Ziyan Tang, Feng Wang

**Affiliations:** 1 Department of Ophthalmology, The First Hospital of Harbin Medical University, Harbin, China

**Keywords:** long-chain non-coding RNA, retinal pigment epithelial cells, epithelial-mesenchymal transition

## Abstract

Regulation of long-chain non-coding RNA01592 (LINC01592) in the process of transforming retinal pigment epithelial (RPE) cells into mesenchymal cells following induction by transforming growth factor beat 1 (TGF-β1) was investigated by interfering with LINC01592 expression in human RPE (hRPE) cells. LINC01592 expression in hRPE cells was significantly increased following treatment with 10 ng/mL TGF-β1 for 48 h. Expression of E-cadherin and Snail were decreased in hRPE cells following induction with TGF-β1 compared with the control group (*P <* 0.05). Following induction by TGF-β1, expression of E-cadherin, alpha-smooth muscle actin (α-SMA), and Snail were significantly lower in the LINC01592-knockdown group compared with the negative control group (*P <* 0.05). LINC01592 overexpression significantly enhanced the viability, proliferation, and migration of hRPE cells induced by TGF-β1 (*P <* 0.05). Following induction by TGF-β1, E-cadherin expression was significantly decreased and α-SMA and Snail expression were significantly increased in the LINC01592-overexpression group compared with the negative control group (*P <* 0.05). RPE cells induced by TGF-β1 exhibited epithelial-mesenchymal transition (EMT). Inhibiting LINC01592 expression could significantly reduce TGF-β1-induced EMT of hRPE cells. The regulatory effect of LINC01592 on EMT in hRPE cells induced by TGF-β1 provides a novel treatment for proliferative vitreoretinopathy.

## INTRODUCTION

Proliferative vitreoretinopathy (PVR), a complication associated with retinal detachment surgery and trauma [1, 2], occurs when simulation and migration of retinal pigment epithelial (RPE) cells is induced by cytokines and oxidative stress, which eventually leads to visual impairment and blindness [3, 4]. Epithelial-mesenchymal transformation (EMT) plays an indispensable role in oxidative stress, stem cell differentiation, growth, and wound healing, but also promotes the occurrence and development of cell fibrosis and cancer pathologies [5, 6]. Previous studies confirmed that EMT of RPE cells mediated by transforming growth factor beta 1 (TGF-β1) is the main reason for pathological changes associated with PVR [7, 8]. RPE cells convert from an epithelial to mesenchymal phenotype and participate in EMT [9, 10]. The transformation of cell differentiation is mediated by key transcription factors such as Snail, a zinc-finger box binding protein and basic helix transcription factor [11].

In addition, long noncoding RNA (lncRNA) such as lnc-ATB, linc-RoR, and HOTAIR have been shown to induce EMT in tumor epithelial cells [12, 13]. Moreover, studies have shown that lncRNA plays an important role in triggering EMT in epithelial cells during tumor metastasis [14, 15]. MALAT1 can promote the proliferation, migration, and epiretinal membrane formation of RPE cells in PVR [16, 17]. In addition, it was confirmed that downregulation of MALAT1 could inhibit the induction of EMT by TGF-β1 in ARPE-19 cells, and significantly reduced the upregulation of EMT-related transcription factors Snail, SLUG, and ZEB1 in RPE cells [18, 19].

Results of our previous lncRNA microarray analysis showed that LINC01592 expression was significantly increased in hRPE cells following induction by TGF-β1 compared with the control group, indicating an important role for LINC01592 in regulation of hRPE cell proliferation. In the present study, we investigated the role of LINC01592 in the process of TGF-β1-induced EMT in hRPE cells.

## RESULTS

### TGF-β1 induced EMT in hRPE cells

**Figure 1:**
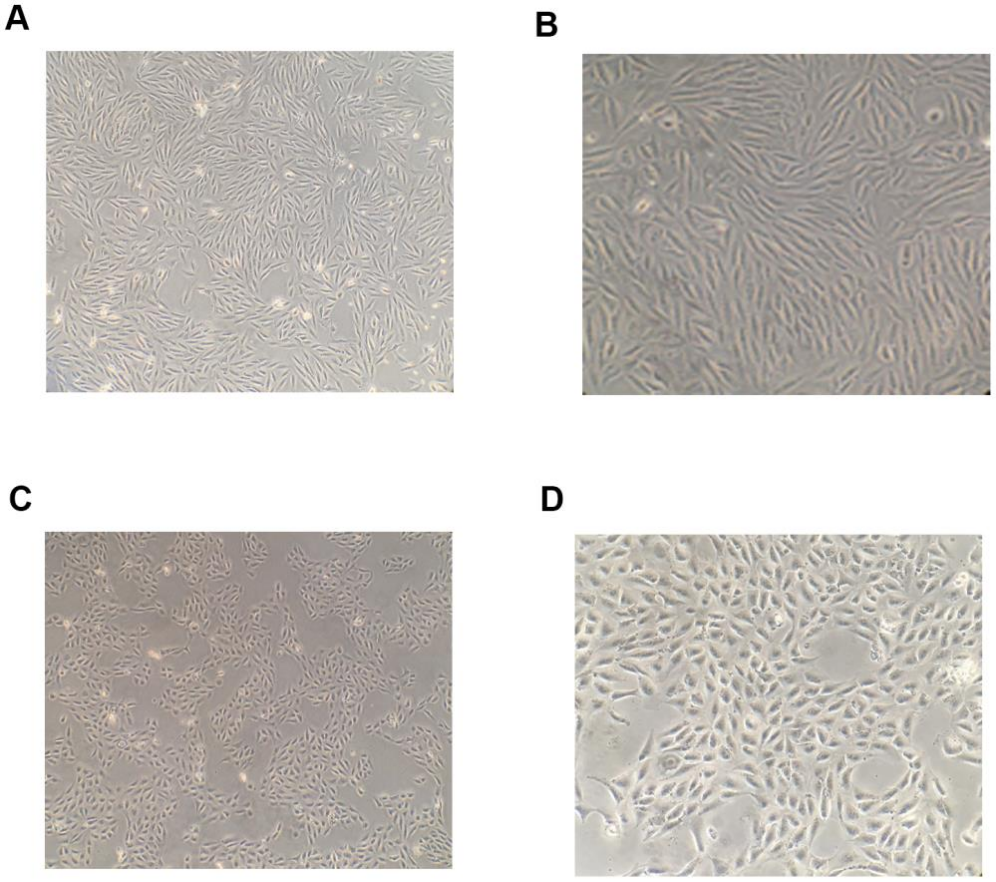
**Morphological changes of RPE cells following induction by TGF-β1.** After 48-h induction with 10 ng/mL TGF-β1, the shape of RPE cells became spindle-like and their arrangement was observed to become loose under a microscope (**A**, **B**) The shape of RPE cells in the blank control group under a microscope (**C**, **D**).

After 48-h intervention with 10 ng/mL TGF-β1, hRPE cells were transformed into loosely arranged spindle-shaped cells, indicating their transition from an epithelial to mesenchymal phenotype (Figure 1).

**Figure 2:**
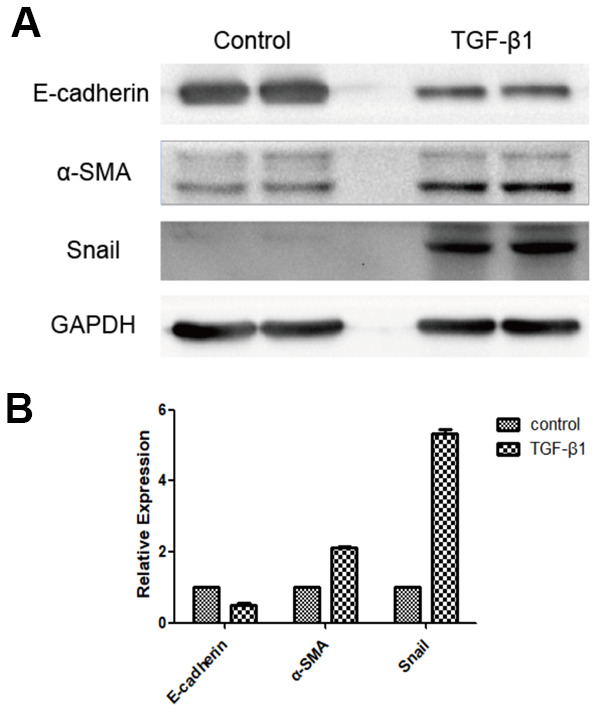
**Expression of EMT marker proteins following induction of RPE cells with TGF-β1, as detected by western blot.** E-cadherin expression was downregulated, whereas expression of α-SMA and Snail were upregulated in the group induced by TGF-β1 (**A**, **B**). The difference of expression levels between the two groups was statistically significant (*P <* 0.05).

Expression of E-cadherin, alpha-smooth muscle actin (α-SMA), and Snail (an EMT-related transcription factor) was decreased in hRPE cells of the experimental group compared with the control group following induction with TGF-β1 for 48 h (*P* < 0.05, Figure 2).

### TGF-β1 induced LINC01592 expression in hRPE cells

**Figure 3:**
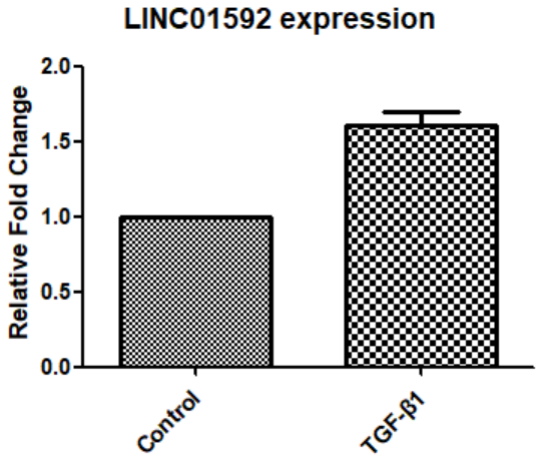
**Expression of LINC01592 was increased following induction of RPE cells by TGF-β1, as detected by RT-PCR.** Expression of LINC01592 mRNA was increased following induction with TGF-β1. The difference in expression levels between the two groups was statistically significant (*P <* 0.05).

Our results show that LINC01952 expression in hRPE cells was significantly increased after 48-h intervention with TGF-β1 compared with the control group (*P* < 0.05, Figure 3).

### LINC01592 knockdown inhibited TGF-β 1-induced EMT in hRPE cells

**Figure 4:**
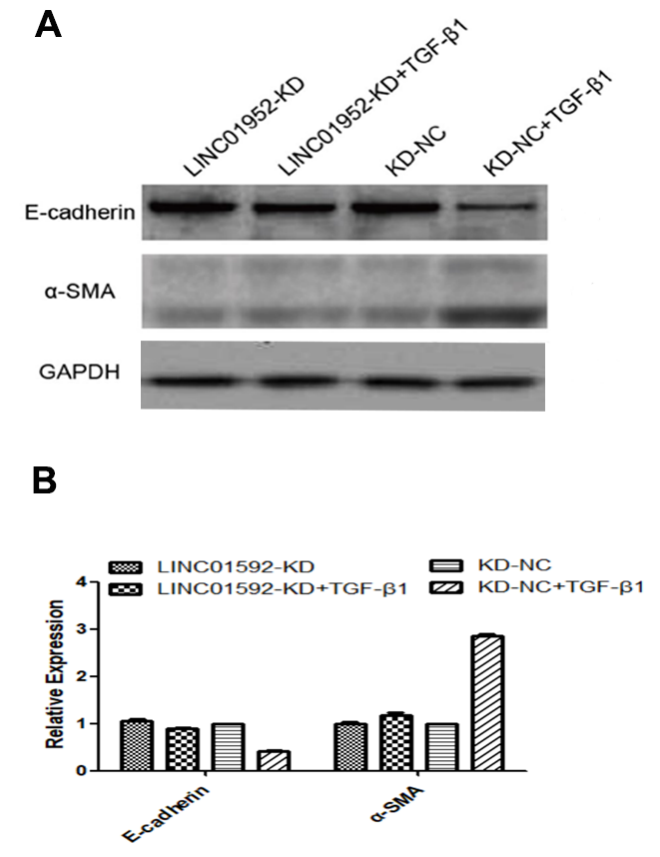
**LINC01592 knockdown reduced TGF-β1-induced EMT of RPE cells.** Expression of EMT molecular makers E-cadherin and α-SMA was detected by western blot. (**A**) Differences in expression levels between groups were statistically significant (**B**, *P <* 0.05).

Our results indicate significantly decreased E-cadherin expression and significantly increased α-SMA expression in hRPE cells of the TGF-β1 group (*P* < 0.05). In addition, LINC01592 expression was increased in the TGF-β1 group (*P* < 0.05). Expression of E-cadherin in the LINC01592-knockdown plus TGF-β1 induction (LINC01592-KD + TGF-β1) group was significantly lower (*P* < 0.05) than in the negative control plus TGF-β1-induction (LINC01592-KD-NC + TGF-β1) group, which also exhibited significantly increased α-SMA expression (*P* < 0.05). These results indicate that downregulation of LINC01592 inhibited the decrease of E-cadherin and increase of α-SMA in hRPE cells induced by TGF*-*β1, which suggests that TGF-β1-induced EMT of hRPE cells can be inhibited (Figure 4).

### LINC01592 knockdown inhibited TGF-β1-induced upregulation of the EMT-related transcription factor Snail

**Figure 5:**
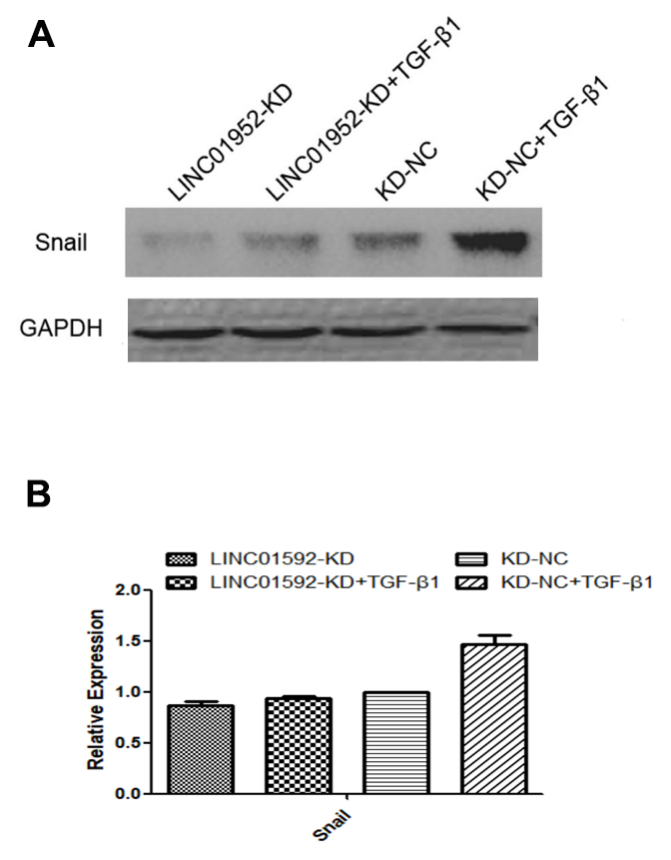
**LINC01592 knockdown decreased TGF-β1-induced upregulation of the EMT-related transcription factor Snail.** Expression of the EMT-related transcription factor Snail was detected by western blot. The difference of expression levels between groups was statistically significant (**A**, **B**; *P <* 0.05).

Expression of Snail, an EMT-related transcription factor, was significantly increased in both LINC01592-KD + TGF-β1 and LINC01592-KD-NC + TGF-β1 groups (*P* < 0.05), but was significantly lower in the LINC01592-KD + TGF-β1 group compared with the LINC01592-KD-NC + TGF-β1 group (*P* < 0.05). These results indicate that inhibition of LINC01592 expression could inhibit Snail expression in hRPE cells following induction by TGF-β1 (*P* < 0.05, Figure 5).

### LINC01592 knockdown reduced proliferation and migration of hRPE cells

**Figure 6:**
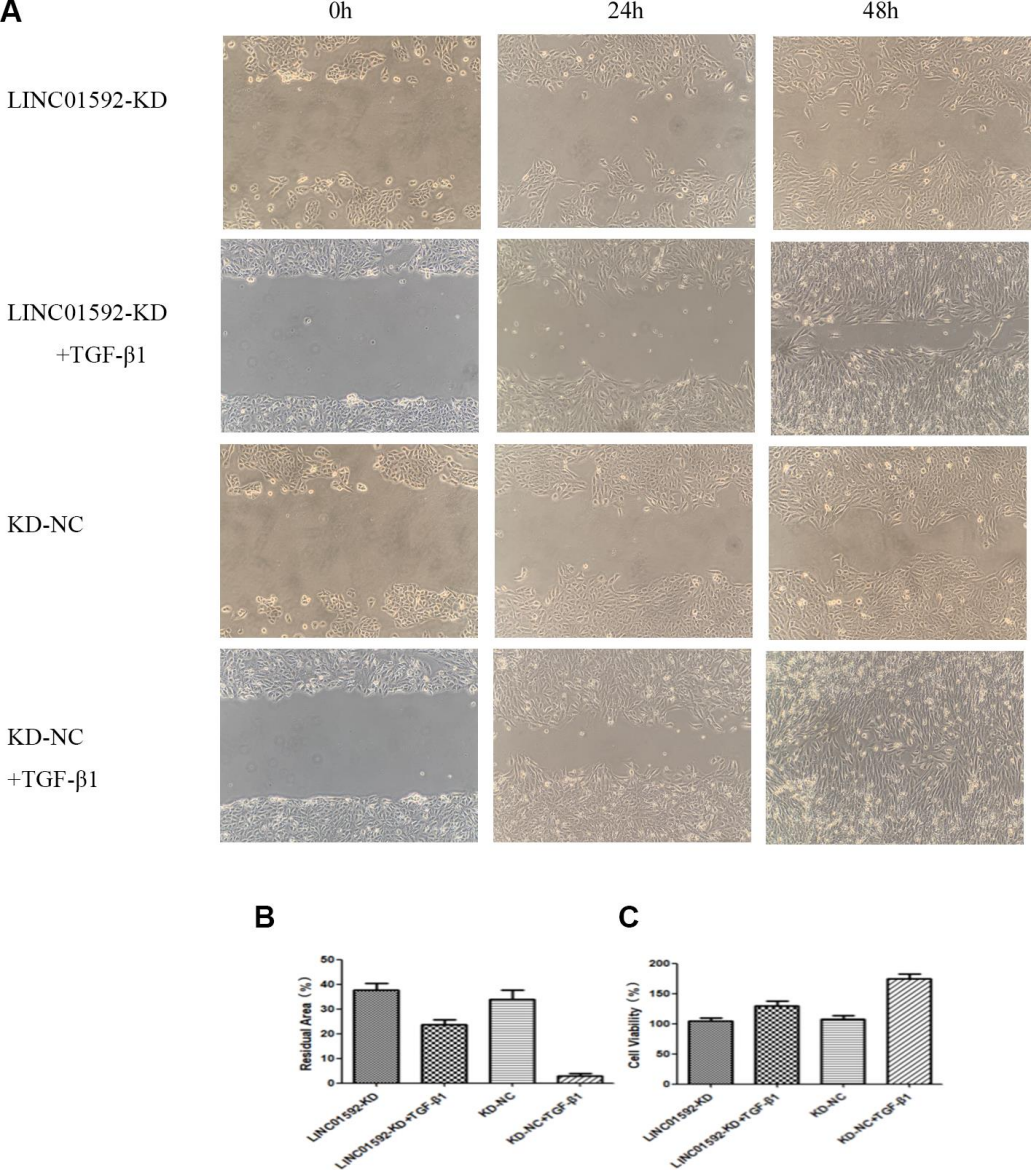
**Knockdown of LINC01592 decreased the proliferation and migration of RPE cells.** Migration of RPE cells in each group was observed under a microscope at 0, 24, and 48 h (**A**). The residual scratch area of RPE cells in each group after transfection and induction by TGF-β1 for 48 h (**B**). Viability of RPE cells was detected by CCK-8 after transfection and induction by TGF-β1 for 48 h (**C**). The difference of expression levels between groups was statistically significant (*P <* 0.05).

The results of cell migration-scratch testing indicated no significant difference between LINC01592-KD and LINC01592-KD-NC groups (*P* > 0.05). However, the residual scratch area of both LINC01592-KD + TGF-β1 and LINC01592-KD-NC + TGF-β1 groups was significantly lower compared with LINC01592-KD and LINC01592-KD-NC groups (*P* < 0.05), respectively. Moreover, the residual scratch area of the LINC01592-KD-NC + TGF-β1 group was significantly lower compared with the LINC01592-KD-NC group (*P* < 0.05). The residual scratch area of the LINC01592-KD-NC + TGF-β1 group was significantly lower compared with the LINC01592-KD + TGF-β1 group (*P* < 0.05). These results suggest that following induction by TGF-β1, proliferation and migration of hRPE cells was decreased in response to reduced LINC01592 expression (Figure 6). Cell Counting Kit 8 (CCK-8) results revealed no significant difference in viability between cells in LINC01592-KD and LINC01592-KD-NC groups (*P* > 0.05). Moreover, no significant differences were observed among LINC01592-KD + TGF-β1, LINC01592-KD-NC + TGF-β1, and LINC01592-KD-NC + TGF-β1 groups (*P* > 0.05). Compared with the LINC01592-KD-NC group, both LINC01592-KD and LINC01592 groups exhibited significantly higher cell viability (*P* < 0.05). Viability of the LINC01592-KD + TGF-β1 group was significantly lower than that of the LINC01592-KD-NC + TGF-β1 group (*P* < 0.05). These results suggest that following induction by TGF-β1, viability of hRPE cells was decreased in response to reduced LINC01592 expression (Figure 6).

### LINC01592 overexpression enhanced TGF-β1-induced EMT of hRPE cells

**Figure 7:**
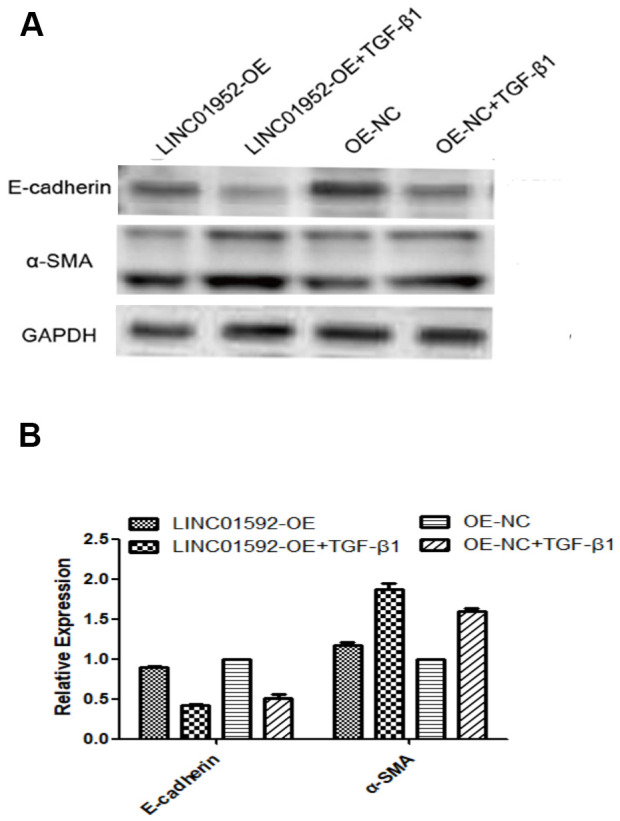
**LINC01592 overexpression enhanced the EMT of hRPE cells induced by TGF-β1.** Expression of EMT molecular makers E-cadherin and α-SMA were detected by western blot. (**A**) The difference in expression levels between groups was statistically significant (**B**, *P <* 0.05).

Our results show that compared with LINC01592 overexpression (LINC01592-OE), negative control (LINC01592-OE-NC), LINC01592 overexpression plus TGF-β1 induction (LINC01592-OE + TGF-β1), and LINC groups, E-cadherin expression was significantly decreased and α-SMA expression was increased in the negative control plus TGF-β1 induction (LINC01592-OE-NC + TGF-β1) group (*P* < 0.05). However, E-cadherin expression was significantly decreased and α-SMA expression was significantly increased in the LINC01592-OE + TGF-β1 group compared with the LINC01592-OE-NC + TGF-β1 group (*P* < 0.05). These results suggest that LINC01592 overexpression could promote downregulation of E-cadherin and upregulation of α-SMA in hRPE cells following induction by TGF-β1, thus enhancing TGF-β1-induced EMT of hRPE cells (Figure 7).

### LINC01592 overexpression enhanced TGF-β1-induced upregulation of the EMT-related transcription factor Snail

**Figure 8:**
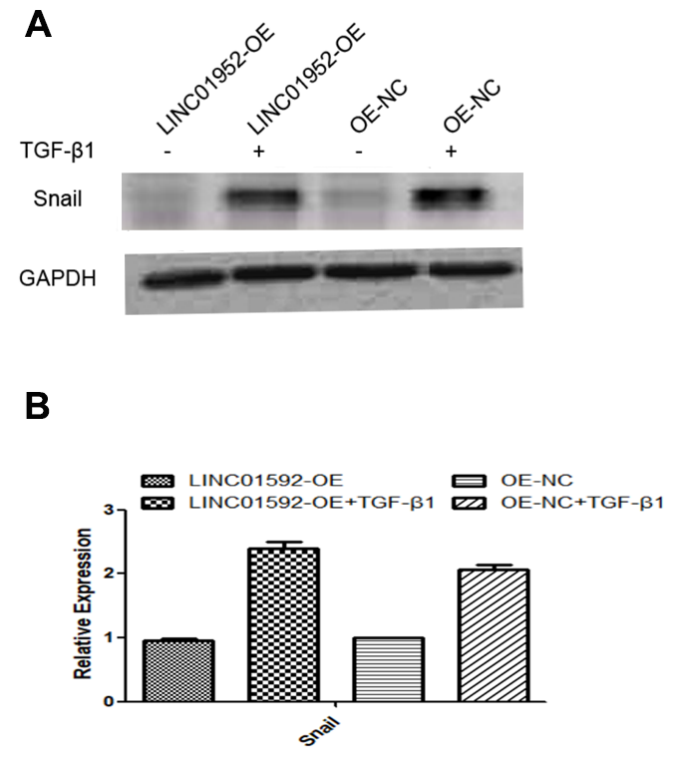
**LINC01592 overexpression increased TGF-β1 induced upregulation of the EMT-related transcription factor Snail.** Expression of the EMT-related transcription factor Snail was detected by western blot. The difference in expression levels between groups was statistically significant (**A**, **B**; *P <* 0.05).

Compared with LINC01592-OE and LINC01592-OE-NC groups, expression of the EMT transcription factor Snail in LINC01592-OE + TGF-β1 and LINC01592-OE-NC + TGF-β1 groups was significantly increased (*P* < 0.05), respectively; however, Snail expression in the LINC01592-OE + TGF-β1 group was significantly higher compared with the LINC01592-OE-NC + TGF-β1 group (*P* < 0.05). Thus, LINC01592 overexpression could promote expression of the EMT transcription factor Snail in hRPE cells following induction by TGF-β1 (Figure 8).

### LINC01592 overexpression enhanced the proliferation and migration of hRPE cells

The results of cell migration-scratch testing indicated no significant difference in residual scratch area between LINC01592-OE and LINC01592-OE-NC groups (*P* > 0.05). However, the residual scratch areas of LINC01592-OE + TGF-β1 and LINC01592-OE-NC + TGF-β1 groups were significantly lower compared with LINC01592-OE and LINC01592-OE-NC groups, respectively (*P* < 0.05). Moreover, the residual scratch area of the LINC01592-OE + TGF-β1 group was significantly lower compared with the LINC01592-OE-NC + TGF-β1 group (*P* < 0.05). These results suggest that LINC01592 overexpression enhanced the proliferation and migration of hRPE cells following induction by TGF-β1.

**Figure 9:**
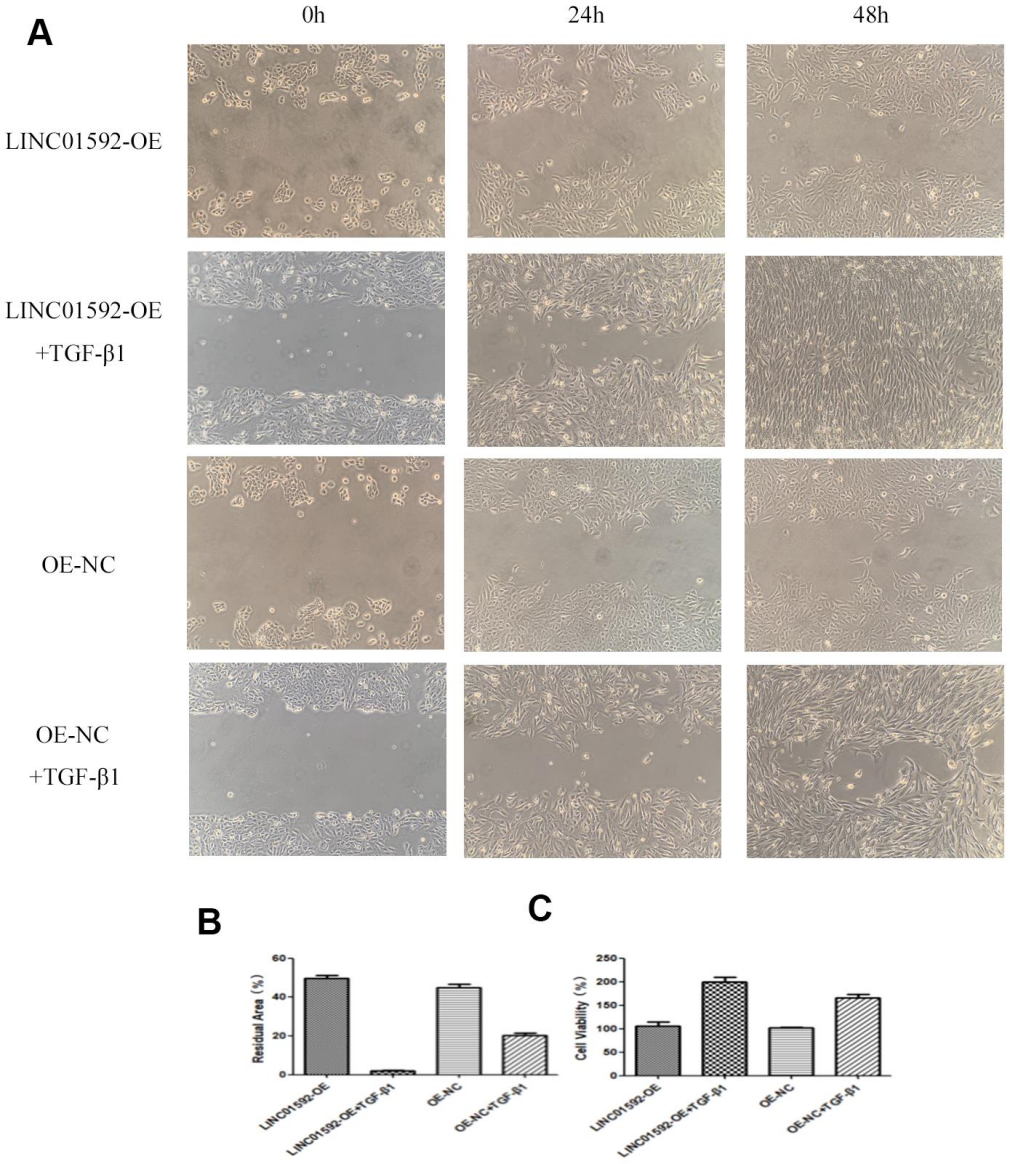
**LINC01592 overexpression increased the proliferation and migration of RPE cells.** Migration of RPE cells in each group was observed at 0, 24, and 48 h (**A**). The residual area of scratch space for RPE cells in each group after transfection and induction by TGF-β1 for 48 h (**B**). Viability of RPE cells was detected by CCK-8 after transfection and induction by TGF-β1 for 48 h (**C**). The difference in expression levels between groups was statistically significant (*P <* 0.05).

CCK-8 assay results indicated no significant difference in cell viability between LINC01592-OE and LINC01592-OE-NC groups (*P* > 0.05). However, compared with LINC01592-OE-NC and LINC01592-OE-NC groups, cell viability of the LINC01592-OE + TGF-β1 group was significantly increased (*P* < 0.05). Importantly, viability of the LINC01592-OE+TGF-β1 group was significantly higher compared with LINC01592-OE and LINC01592-OE-NC groups (*P* < 0.05). These results suggest that overexpression of LINC01592 increased the viability of hRPE cells following induction by TGF-β1 (Figure 9).

## DISCUSSION

PVR is a type of ocular fibrous disease characterized by the formation of a contractile epiretinal membrane, the main cellular component of which is RPE cells. EMT occurs when RPE cells detach from the damaged retina and migrate into the vitreous cavity or subretinal space, whereby they are stimulated by various cytokines [20–23]. After RPE cells acquire a mesenchymal phenotype, their migration, invasiveness, and anti-apoptotic ability are enhanced, and they begin to produce extracellular matrix [24–27]. RPE cells that undergo the EMT process change from epithelial cells to fibroblast-like cells and participate in the formation of an epiretinal membrane [28–30]. Following retinal damage, TGF-β1 released from vitreous or serum is the main factor stimulating EMT in RPE cells [31]. Although EMT has been confirmed as the main pathogenic factor of PVR in RPE cells, the mechanism by which EMT occurs RPE cells remains unclear.

In the present study, expression of E-cadherin was decreased but that of α-SMA and Snail was increased following TGF-β1 induction. These results confirmed that EMT could occur in RPE cells 48 h after TGF-β1 intervention.

LINC01592 is a 2367-bp lncRNA located in two bands of the 13 region of chromosome 8. In this study, RNA was extracted from hRPE cells treated with TGF-β1 for 48 h. RT-PCR assay results confirmed that LINC01592 expression was significantly increased in RPE cells treated with TGF-β1, suggesting the potential involvement of LINC01592 in regulation of EMT in hRPE cells during the development of PVR.

Our results suggest that inhibiting LINC01592 expression not only inhibited TGF-β-induced EMT of hRPE cells but also reduced their proliferation and migration. In addition, increased expression of the EMT-related transcription factor Snail induced by TGF-β1 was inhibited by knockdown of LINC01592 expression. Previous studies implicated Snail in some signaling pathways associated with EMT, which suggests that LINC01952 may regulate EMT in RPE cells by participating in a signaling pathway involving Snail. However, the specific signaling pathways affected by LINC01592 and Snail in TGF-β1-induced EMT of hRPE cells require further study.

Following induction by TGF-β1, LINC01592 overexpression could promote the EMT of hRPE cells and enhance their proliferation and migration ability. In addition, LINC01592 overexpression enhanced expression of the EMT transcription factor Snail following induction by TGF-β1. These results indicate that LINC01592 not only participated in the process of EMT in hRPE cells induced by TGF-β1 but also regulated their proliferation and migration and promoted the EMT process.

Following EMT of RPE cells, they produce and participate in the formation of an epiretinal membrane – the main pathogenic factor of PVR. In this study, TGF-β1 was used to induce EMT in hRPE cells. The effect of lncRNA on EMT, proliferation, and migration of hRPE cells was confirmed by interfering with LINC01592 expression. Reducing LINC01592 expression could inhibit the EMT process of hRPE cells following induction by TGF-β1, thus realizing the possibility of inhibiting the occurrence and development of PVR. In addition, we found that LINC01592 may regulate EMT in hRPE cells by participating in a signaling pathway involving the transcription factor Snail. TGF-β1 promoted EMT of hRPE cells; LINC01592 could regulate the process of TGF-β1-induced EMT of hRPE cells, and reduced expression of LINC01592 inhibited the EMT process. The regulatory effect of LINC01592 on TGF-β1-induced epithelial interstitialization of hRPE cells may involve signaling pathways involving Snail.

Our findings confirm that LINC01592 is related to the occurrence and development of PVR. At present, no report has described the mechanism by which LINC01592 participates in the pathogenesis of EMT in hRPE cells. To provide a new target for gene therapy of PVR, the present study elucidated the role of LINC01592 in the process of TGF-β1-induced EMT in hRPE cells.

## MATERIALS AND METHODS

All procedures of this experiment were approved by the First Affiliated Hospital of Harbin Medical University (Harbin, China) ethics committee and conformed with Association for Research in Vision and Ophthalmology guidelines for ophthalmic and vision studies.

### EMT of hRPE cells following induction by TGF-β1

Donated eyeballs were from the eye bank of First Affiliated Hospital of Harbin Medical University. hRPE cells were carefully collected and then treated with 0.25% trypsin for 1 h. hRPE cells were inoculated in six-well plates and cultured in an incubator at 37° C and 5% CO_2_ for 12 h, until the cells completely adhered. hRPE cells were used for experiments after they reached confluence.

TGF-β1 dry powder (Sigma-Aldrich, St. Louis, MO, USA) was centrifuged and dissolved in Dulbecco’s Modified Eagle’s Medium with F-12 Nutrients (DMEM/F12) to prepare a 60-ng/mL solution. After culturing cells in serum-free medium (Corning, Corning, NY, USA) for 12 h, 10 ng/mL TGF-β1 solution was added to the cells.

### Transfection of hRPE cells with LINC01592

LINC01592-knockdown (LINC01592-KD), LINC01592-overexpression (LINC01592-OE), and their respective negative control plasmids (KD-NC and OE-NC, respectively) were provided by Jikai Gene Chemical Technology (Shanghai, China). The LINC01592 knockdown plasmid had a target sequence of GCCTATTGTTATTGGGCAT in the hU6-MCS-CMV-GFP-SV40-Neomycin vector. The LINC01592 overexpression plasmid was CMV-MCS-IRES-EGFP-SV40-Neomycin.

hRPE cells were divided into LINC01592-KD, LINC01592-KD + TGF-β1, KD-NC, and KD-NC + TGF-β1 groups. Overexpression experiment groups included LINC01592-OE, LINC01592-OE + TGF-β1, OE-NC, and OE-NC+TGF-β1 groups. LINC01592-KD, KD-NC, LINC01592-OE, and OE-NC groups were generated by incubating 3 × 10^5^ RPE cells in DMEM/F12 medium containing 10% fetal bovine serum (FBS) at 37° C, 5% CO_2_ for 24 h. For transfection, the appropriate plasmid and Lipofectamine 2000 (Invitrogen, Carlsbad, CA, USA) were added Opti-MEM serum-free culture medium. DNA (μg) and Lipofectamine 2000 (μL) were mixed at a ratio of 1:2.5 at room temperature for 20 min. After discarding the original culture medium, the DNA-Lipofectamine 2000 mixture was added such that each well of the six-well plate contained 4 μg of plasmid and 10 μL of Lipofectamine 2000. Transfection efficiency of hRPE cells was observed by fluorescence microscopy.

### Western blot

Cell debris and lysates were centrifuged at 12000 r/min for 15 min. After collecting the supernatant, the protein concentration was determined according to the instructions of a bicinchoninic acid assay kit. A 12% gel was prepared and 30 μg of protein was loaded into each lane. Proteins were subsequently transferred to polyvinylidene fluoride membranes, which were blocked in 5% skimmed milk powder in phosphate-buffered saline containing Tween 20 (PBS-T), placed on a horizontal shaker, and sealed for 1 h. Next, membranes were incubated with mouse anti-human E-cadherin (1:1000; Santa Cruz Biotechnology, Dallas, TX, USA), rabbit anti-human α-SMA (1:500, Santa Cruz Biotechnology), rabbit anti-human Snail (1:1000, Santa Cruz Biotechnology), and/or rabbit anti-human GAPDH (1:1000, Santa Cruz Biotechnology) antibodies at room temperature for 2 h, followed by 4° C for 12 h. Subsequently, membranes were washed three times (10 min each) with PBS-T on a decolorizing shaking bed, followed by incubation with appropriate secondary antibodies in a horizontal shaking bed at room temperature for 1 h. Membranes were analyzed according to instructions of an enhanced chemiluminescence kit (Bio-Rad, Hercules, CA, USA) [32].

### Real-time quantitative polymerase chain reaction (RT-qPCR)

RNA extracts were treated with RNase-Free H_2_O. After discarding the culture medium from six-well plates, cells were washed twice with PBS at 4° C. Next, 150 μL of RNA was added to each well along with extract Buffer R-I from the kit, and the mixture was pipetted up and down 8–10 times. The mixture containing cell debris and lysate was transferred to a 1.5-mL centrifuge tube. RNA was extracted according to the instructions of an RNA extraction kit (Invitrogen).

After thermal denaturation of RNA at 65° C for 5 min, RNA was immediately cooled on ice. The reaction liquid (4 μL of 4× DN Master Mix, 1 μg of RNA template, 11 μL of Nuclease-free Water) was stirred gently and evenly, then incubated at 37° C for 5 min. Reverse transcription was carried out and reactions were prepared on ice as follows: 4 μg/L of 4× DN Master Mix, 1 μg of RNA template, 11 μg/L Nuclease-free Water, and 4 μg/L of 5× RT Master Mix II. Reactions were carried out at the following temperatures: 37° C for 15 min, 50° C for 5 min, 98° C 5 min, and then maintained at 4° C. The DNA solution was stored at -20° C after the reaction. Subsequently, reactions containing 6.4 μL of sterilized distilled water, 6 pmol/0.6 μL of forward primer, 6 pmol/0.6 μL of reverse primer, 0.4 μL of 50× ROX reference dye, and 2 μL of DNA solution were prepared on ice. PCR was carried out under the following conditions: pre-denaturation at 95° C 60 s, denaturation at 95° C 15 s, extension at 60° C 30 s, and final extension at 60° C for 60 s (a total of 40 cycles).

Primer sequences were as follows: LINC01592 forward 5ʹ-AGG GCT CAG TAG ATT TGC CC-3ʹ, LINC01592 reverse 5ʹ-CAC CTA ACG GAA ATG TCG GC-3ʹ, GAPDH forward 5ʹ-CGA GAT CCC TCC AAA ATC AA-3ʹ, and GAPDH reverse 5ʹ-TTC ACA CCC ATG ACG AAC AT-3ʹ [33].

### Cell migration-scratch test

hRPE cells (3 × 10^5^ per well of six-well plate) were inoculated in DMEM/F12 medium containing 10% FBS at 37° C and 5% CO_2_ for 24 h. A zigzag scratch was made perpendicular to the plate orifice. Next, cells were washed twice with PBS. Wells were divided into LINC01592-KD + TGF-β1, KD-NC + TGF-β1, LINC01592-OE + TGF-β1, and OE-NC + TGF-β1 groups. The concentration of TGF-β1 was adjusted to 10 ng/mL and a constant volume of 2 mL was added in serum-free medium for LINC01592-KD, KD-NC, LINC01592-OE, and OE-NC groups. Samples were analyzed after 0, 24, and 48 h of incubation at 37° C with 5% CO_2_.

### CCK-8 analysis

hRPE cells were divided into LINC01592-KD, LINC01592-KD + TGF-β1, KD- NC, KD-NC + TGF-β1, LINC01592-OE, LINC01592-OE + TGF-β1, OE-NC, and OE-NC + TGF-β1 groups. All procedures were performed according to the CCK-8 kit manufacturer’s instructions (Abcam, Cambridge, UK). hRPE cells were incubated with DMEM/F12 medium containing 10% FBS in 96-well plates at 37° C and 5% CO_2_ for 24 h. A mixture of 0.2 μg plasmid and 0.5 μg Lipofectamine 2000 was added to each well. The control group was treated with100 μL of Opti-MEM serum-free culture medium. Serum-free medium and 10 ng/mL TGF-β1 solution were added to wells of LINC01592-KD + TGF-β1, KD-NC + TGF-β1, LINC01592-OE + TGF-β1, and OE-NC + TGF-β1 groups, which were incubated at 37° C and 5% CO_2_ for 48 h. Next, 10 mL of CCK-8 solution and 90 mL of complete culture medium were added to plate for incubation at 37° C and 5% CO_2_ for 2 h. The absorbance at 450 nm was measured by enzyme labeling.

Cell viability was calculated using the following formula: Cell viability (%) = [A (medication) - B (blank)]/[C (0 medication) - B (blank)] × 100, whereby A is the absorption of experimental group wells with cells, CCK-8 solution, and culture medium, following transfection with plasmid and intervention with or without TGF-β1; B is the absorption of wells with CCK-8 solution and culture medium, but without cells; and C is the absorption of control group wells with cells, CCK-8 solution, and culture medium, but without plasmid transfection or TGF-β1 intervention of [34]. All experiments were repeated three times.

### Statistical analysis

SPSS22.0 software was used for statistical analysis. The data were analyzed by one-way ANOVA and double-tailed t-test.
